# Production of Volatile and Sulfur Compounds by 10 *Saccharomyces cerevisiae* Strains Inoculated in Trebbiano Must

**DOI:** 10.3389/fmicb.2016.00243

**Published:** 2016-03-04

**Authors:** Francesca Patrignani, Fabio Chinnici, Diana I. Serrazanetti, Pamela Vernocchi, Maurice Ndagijimana, Claudio Riponi, Rosalba Lanciotti

**Affiliations:** ^1^Department of Agricultural and Food Sciences, University of BolognaBologna, Italy; ^2^Interdepartmental Centres for Industrial Research, University of BolognaCesena, Italy; ^3^Human Microbiome Unit, Genetic and Rare Diseases Area, Bambino Gesu Research Hospital IRCCSRome, Italy; ^4^Department of Agricultural Food and Nutritional Science, Agriculture/Forestry Centre, University of AlbertaEdmonton, AB, Canada

**Keywords:** *Saccharomyces cerevisiae*, sulfur compounds, volatile compounds, Trebbiano wine, electronic nose

## Abstract

In wines, the presence of sulfur compounds is the resulting of several contributions among which yeast metabolism. The characterization of the starter *Saccharomyces cerevisiae* needs to be performed also taking into account this ability even if evaluated together with the overall metabolic profile. In this perspective, principal aim of this experimental research was the evaluation of the volatile profiles, throughout GC/MS technique coupled with solid phase micro extraction, of wines obtained throughout the fermentation of 10 strains of *S. cerevisiae*. In addition, the production of sulfur compounds was further evaluated by using a gas-chromatograph coupled with a Flame Photometric Detector. Specifically, the 10 strains were inoculated in Trebbiano musts and the fermentations were monitored for 19 days. In the produced wines, volatile and sulfur compounds as well as amino acid concentrations were investigated. Also the physico-chemical characteristics of the wines and their electronic nose profiles were evaluated.

## Introduction

The wine flavor and aroma are the result of several interactions between a huge amount of chemical compounds and sensory receptors. The wine flavor can be the sum of varietal (deriving from the grapes), pre-fermentative (deriving from grape crushing and must conditioning), fermentative (generated during fermentations by yeasts and/or bacteria), and post-fermentative flavors (generated by the wood release or the chemical transformation during conservation; Swiegers et al., 2005).

However, volatiles from fermentation largely dominate wine flavor, since yeasts metabolize grape sugars and other components into ethanol, carbon dioxide, and hundreds of secondary end-products, contributing to the wine character (Fleet, 2003). The aroma compounds from yeast metabolisms are constituted by higher alcohols, esters, organic acids and aldehydes (Lambrechts and Pretorius, 2000; Vernocchi et al., 2011, 2015). The amount of these compounds constitutes the overall expression of the fermentative flavor and, if in excess, some of them (i.e., acetic acid, acetaldehyde) may also be regarded as undesirable (Liu and Pilone, 2000; Styger et al., 2011). Also sulfur compounds, which can be considered a “double-edged sword,” can contribute positively or negatively to wine aroma (Vichi and Cortes-Francisco, 2015). Positive examples are furfurylthiol (roast coffee' aroma) (Tominaga et al., 2000) and the “fruity” polyfunctional thiols 3-mercaptohexan-1-ol (3MH), 4-mercapto-4-methyl-pentan-2-one (4MMP), and 3-mercaptohexyl acetate (3MHA), that impart passion fruit, grapefruit, gooseberry, guava, and “box hedge” aromas (Swiegers et al., 2005; Swiegers and Pretorius, 2007). In particular, these thiols affect the distinctive sensory characteristics of wines made from the grape variety Sauvignon Blanc (Harsch and Gardner, 2013). On the contrary, the highly volatile sulfur compounds (HVSC) have a negative impact in wine because, with their low odor threshold (in the order of ppb), they imparts a powerful odor described as soup-like, meaty, boiled potato, rotten egg-like off-flavor, and cooked cabbage aroma (Vermeulen and Gus, 2005; Davis and Qian, 2011; Franco-Luesma and Ferreira, 2014). Commonly found HVSCs include methanethiol, dimethyl sulphide (DMS), dimethyl disulphide (DMDS), dimethyl trisulphide (DMTS), 3-methylthio-1-propanal (methional), 3-methylthio-1-propanol (methionol), and *S*-methylthioesters of short-chain fatty acids (acetate, propanoate, and butanoate). Also the production of H_2_S represents in winemaking a global problem, resulting in a loss of wine quality and a rejection from the consumers. During alcoholic fermentation, *Saccharomyces cerevisiae* can be responsible for the production of several sulfur compounds *via* the sulfate reduction pathway (Swiegers and Pretorius, 2007), but the majority of H_2_S produced during winemaking occurs as a result of the biosynthesis of the sulfur containing amino acids, methionine, and cysteine, which occur in low concentrations in grape juice, through the sulfate reduction sequence (SRS). However, for sulfides as for all the other classes of volatile compounds, the yeast strain used for fermentation is the main factor influencing their production (Rainieri and Pretorius, 2000; Fleet, 2008). However, their perception in wine is related to the other volatile compounds, and it is also the result of the interaction with non-volatile molecules. Because sulfur compounds are present in wine at very low concentrations, they are usually determined by gas-chromatographic techniques and their detection represents a methodological challenge. Headspace techniques are generally preferred due to the high volatility of these compounds and their relatively low solubility in organic solvents. Consequently, simple static headspace or headspace solid phase microextraction are generally used for their extraction and the sulfur chemiluminiscence (SCD) and the flame photometric detectors (FPD) for their detection (Franco-Luesma and Ferreira, 2014).

Thus, the main goal of this research was characterize 10 strains of *S. cerevisiae*, endowed for good oenological properties, producers of H_2_S in strain dependent way, also for the production of HVSC, by using a suitable and reliable technique, since they are fundamental for the sensory wine features but deeply investigated. Because, as previously underlined, the wine volatile profiles are the outcome of the ratio and interaction of several molecules (volatile and not), also the production of volatile compounds and electronic nose profiles were investigated to evaluate the strain volatile fingerprinting and their effects on wine features. In order to verify their potential use for the production of Trebbiano wine, the strains were inoculated in Trebbiano must determining also the fermentation kinetics and the aminoacidic compositions.

## Materials and methods

### Strains

Ten *S. cerevisiae* strains (L234, L288, L674, L951, M630, M692, U5298, 6944, 7541, 6644), able to produce in strain dependent way H_2,_S belonging to ASTRA srl, Faenza, Italy, were employed in the research (Table 1).

**Table 1:** **Main features of the *Saccharomyces cerevisiae* strains used in the research**.

**Identifier**	**Strain**	**H2S Production**
A	L234	++++^*^
B	L288	+^**^
C	L674	+++^***^
D	L951	+
E	M630	−^****^
F	M692	+++
G	U5298	+++
H	6944	+
I	7541	++
L	6644	−

* Very high producer;

** Low producer;

*** High producer;

**** *no production*.

Before using, the frozen strains were sub-cultured three times in Sabouraud broth medium (Oxoid, Basingstoke, UK) at 28°C for 48 h.

### Micro-vinifications

Grape must of Trebbiano variety (vintage 2011) was used to test the effects of the different strains of *S. cerevisiae* on wine characteristics. Until the use, the must was kept frozen. The Trebbiano must features are reported in Table 2. Before inoculation, the must was flash pasteurized (70°C for 20 s). The fermentations were carried out in 500-ml flasks filled with 400 ml of Trebbiano must. For each strain considered, three different micro-vinifications were performed. Each strain was inoculated at level of about 6 Log cfu ml^−1^. The inoculations were performed using 48-h pre-cultures in the same must. The temperature was kept at 18°C during alcoholic fermentation. The weight lost was used to follow the fermentation process. After the completion of alcoholic fermentation, the different wine samples were separated by filtration.

**Table 2:** **Enological features of Trebbiano wines in relation to the strain used for fermentation**.

**Strain**	**Sugars** **(g l^−1^)**	**SO_2_** **(mg l^−1^)**	**Total acidity (expressed as g l^−1^ of tartaric acid)**	**Succinic acid** **(g l^−1^)**	**Malic acid** **(g l^−1^)**	**Lactic acid** **(g l^−1^)**	**ABV** **(%vol) ABV (%vol)**	**pH** **pH**
Must	227 ± 11^A^	−^*^	9.66 ± 0.25^A^	0.05 ± 0.01^A^	7.49 ± 1.13^A^	0.06 ± 0.02^A^	−^*^	3.21 ± 0.01^A^
A	0.75 ± 0.15^B^	5.0 ± 1.0^A^	6.98 ± 0.15^B^	1.74 ± 0.02^B^	1.90 ± 0.45^B^	0.80 ± 0.10^B^	13.56 ± 0.55^A^	3.18 ± 0.01^B^
B	0.86 ± 0.13^B^	5.0 ± 0.8^A^	5.48 ± 0.21^C^	0.59 ± 0.05^C^	0.56 ± 0.10^C^	0.02 ± 0.0^C^	13.48 ± 0.60^A, B^	3.22 ± 0.02^A^
C	1.03 ± 0.20^BC^	5.0 ± 1.1^A^	5.93 ± 0.18^D^	0.37 ± 0.09^D, E^	0.40 ± 0.08^C, D^	0.07 ± 0.0^A^	13.40 ± 0.45 ^A, B^	3.27 ± 0.01^C^
D	8.58 ± 0.45^D^	8.0 ± 0.5^B^	10.65 ± 0.45^E^	0.19 ± 0.03^F^	0.05 ± 0.01^E^	0.43 ± 0.04^D^	12.70 ± 0.15^C^	3.14 ± 0.01^D^
E	1.57 ± 0.10^E^	8.0 ± 0.7^B^	10.20 ± 1.1^E, A^	1.02 ± 0.09^G^	0.22 ± 0.01^F^	0.61 ± 0.06^E^	13.21 ± 0.25^B^	3.20 ± 0.0^A^
F	2.54 ± 0.25^F^	8.0 ± 1.1^B^	7.20 ± 0.13^B^	0.28 ± 0.03^D^	0.17 ± 0.05^F^	0.35 ± 0.01^F^	13.11 ± 1.12^A, B, C^	3.13 ± 0.02^D, E^
G	3.12 ± 0.18^G^	8.0 ± 1.2^B^	12.00 ± 0.15^F^	0.38 ± 0.04^E^	0.03 ± 0.01^E^	0.07 ± 0.01^A^	12.98 ± 0.95^A, B, C^	3.11 ± 0.01^E^
H	1.62 ± 0.10^E^	10.0 ± 0.9^C^	7.76 ± 0.85^B^	0.94 ± 0.05^G^	1.32 ± 0.03^G^	0.03 ± 0.0^G^	13.18 ± 0.39^A, B, C^	3.05 ± 0.02^F^
I	1.26 ± 0.08^C^	13.0 ± 1.1^D^	5.10 ± 1.10^C, D^	0.72 ± 0.02^H^	0.31 ± 0.04^D^	0.09 ± 0.0^H^	13.38 ± 0.25^A, B^	3.27 ± 0.01^C^
L	3.49 ± 0.10^H^	5.00 ± 0.2^A^	7.91 ± 0.94^B^	0.29 ± 0.01^D^	0.88 ± 0.02^H^	0.02 ± 0.0^C^	13.20 ± 1.01^A, B, C^	3.06 ± 0.02^F^

* *not performed; For each column considered, values with the same superscript letter are not statistically different (P > 0.05)*.

### Chemical analyses

Residual sugars, SO_2_, ethanol, pH, and total acidity were performed according to the Official EU Methods (OJEU, 2010).

### Determination of volatile compound profiles

The volatile molecule profiles of Trebbiano wines were analyzed by solid-phase microextraction coupled with gas chromatography-mass spectrometry (SPME-GC-MS) according to the method of Vernocchi et al. (2015). A polyacrylate-coated fiber (85 μm; Supelco, Bellefonte, PA) and a manual SPME holder (Supelco) were used after preconditioning, according to the manufacturer's guidelines. Before each head-space sampling, the fiber was exposed to the gas chromatograph inlet for 5 min for thermal desorption at 250°C in a blank sample. Five milliliter of wine samples were placed in 10 ml glass vials, with 1 g NaCl and 10 μL 4-methyl-2-pentanol (initial concentration of 10000 mg l^−1^)(Sigma, Milan, Italy) as internal standard. The samples were then heated for 10 min at 45°C. The SPME fiber was exposed to each sample for 40 min. Both the equilibration and absorption phases were carried out under stirring. The fiber was then inserted into the injection port of the gas chromatograph for a 5-min sample desorption. GC–MS analyses were performed on an Agilent 7890A (Agilent Technologies, Palo Alto, CA) coupled to an Agilent 5975C mass selective detector operating in electron ionization mode (ionization voltage 70 eV) and using a Chrompack CP-Wax 52 CB capillary column (50 m, 0.32 mm i.d.; Chrompack, Middelburg, Netherlands). Volatile compounds were separated using helium as carrier gas (1 ml min^−1^). The temperature program was 50°C for 2 min, then programmed at 1.5°C min^−1^ to 65°C, and finally at 4.5°C min^−1^ to 220°C, which was maintained for 20 min. Injector, interface, and ion source temperatures were 250, 250, and 230°C, respectively. Identification of the compounds detected in the wine samples was performed comparing mass spectra of compounds with those contained in an available database (NIST version 2005) and those of pure standards.

### Determination of sulfur compounds

The extraction of sulfur compounds from wine was performed by using the method proposed by Moreira et al. (2002). Briefly, 50 ml of wine were extracted twice with 5 ml of dichloromethane after the addition of 4 grams of sodium sulfate and of 500 μl of i.s. [ethyl (methylthio)acetate] at 500 μg l^−1^ to have a final concentration of 50 μg l^−1^. The two organic phases were mixed and the solution was concentrated to 1/10 under a nitrogen flow. Finally, 2 μL of the extract was injected into the chromatograph.

For the analyses, a gas-chromatograph equipped with a Flame-Photometric-Detector (Clarus 500, Perkinelmer) fitted with a 30 m Elite-5 (Supelco, Bellefonte, PA, USA) (i.d. 0.53 mm) column was used. The identification was based on the comparison of the peak retention times with those of pure standards while the quantification was performed by using calibration curves, obtained with reagents Pure standards (>95%) of methanethiol and ethanethiol from Fluka (Steinheim, Germany), dimethylsufide from Merck (Darmstadt, Germany), sodium sulfide, ethylmethylsulfide, 1-propanethiol, thiophene, diethyldisulfide, dimethyldisulfide, diethylsulfide from Sigma-Aldrich (Steinheim, Germany).

### Determination of the amino acids release in wine

The analysis of amino acids in wine was performed according to the method proposed by Ndagijimana et al. (2010, unpublished data).

One ml of of NaOH 1% was added to 1 ml of standard solution or to 1 ml of freeze dried samples supernatants in a silanized micro reaction vessel and vortexed for 10 s. Two-hundred microliter of the mixture were collected in a new micro reaction vessel, added with methanol and pyridine and vortexed for 10 s in presence of 10 μL of decanoic acid (10.000 ppm—solution in ethanol 70%). The following ratios of aqueous phase/methanol/pyridine was used 6:2.1.

An increasing volume of ECF (18 ul) was then added to the mixture to evaluate the efficiency of the derivatizing agent and the mixture was vortexed for 20 s. The same procedure was repeated twice. In order to extract the derivatized analytes, 400 ul of chloroform were added and the mixture vortexed for 20 s. The control of the pH of the reaction medium was performed by means of addition of 400 ul of sodium bicarbonate 50 mM. In order to remove traces of water, anhydrous sodium sulfate was added then the organic phase was carefully collected in a glass silanized conical tube and subjected to GC/MS analysis. The derivatized extracts (both form culture and from standards) were analyzed with a Agilent 7890 gas chromatograph coupled with a 5973C mass spectrometer (Agilent Technologies, USA). One microliter of the extracts was injected into a SPB5 capillary column coated with 5% diphenyl cross-linked 95% dimethylpolysiloxane (60 m × 250 μm i.d., 0.25-μm film thickness; Supelco, Palo Alto, USA) in the split mode (30:1). Preliminary experiments, described in the results, permitted to choose the subsequent conditions. The injection and interface temperatures were set to 250°C and the ion source temperature was adjusted to 200°C. Initial GC oven temperature was 80°C; 2 min after injection, the GC oven temperature was raised to 140°C with 10°C min^−1^, to 240°C at a rate of 4°C min^−1^, to 280°C with 10°C min^−1^ again, and finally held at 280°C for 3 min. Helium was used as the carrier gas with a flow rate of 1 mL min^−1^. The analyses were performed with electron impact ionization (70 eV) in the full scan mode (m/z 30–550).

The identification of analytes was performed by comparison of their retention times and their mass spectra data with those of pure standards analyzed under the same conditions. Moreover, the retention index of the analytes of interest was calculated by means of results related to a mixture of n-alcanes (C10-C24) analyzed under the GC-MS conditions above described. The following equation was used for the calculation of retention index:

$$\begin{array}{l} RI\left(x\right)=100\times z+100\times \frac{\text{RT}\left(\text{x}\right)-\text{RT}\left(\text{z}\right)}{\text{RT}\left(z+1\right)-\text{ RT}\left(z\right)} \end{array}$$

where *RI(x)* is the retention index of the unknown analyte, z is the number of carbon atoms of the n-alkane eluting before the analyte unknown and (z + 1) is the number of carbon atoms of the n-alkane eluting after the peak of interest, RT(x) is the retention time of analyte unknown, RT(z) is the retention time of the n-alkane eluting before the analyte unknown and RT(z+1) is the retention time of of the n-alkane eluting after the peak of interest. All the GC–MS raw files were converted to netCDF format via Chemstation (Agilent Technologies, USA) and subsequently processed by the XCMS toolbox (http://metlin.scripps.edu/download/). XCMS software allows an automatic and simultaneous retention time alignment, matched filtration, peak detection, and peak matching. The resulting table containing information such as peak index (retention time-m/z pair) and normalized peak area was exported into R (www.r-project.org) for subsequent statistical analysis.

### Determination of electronic nose profiles

The electronic nose profiles of the different Trebbiano wines were recorded using a Pen2 Electronic Nose (Airsense Analytics GmbH, Schwerin, Germany) composed of an array of 10 temperature-moderated metal-oxide sensors (MOS), a sampling system, a data acquisition system, and a data processing system. Each sensor is sensible to different kind of volatile molecules For the analysis, 5 ml of wine sample was placed in 40 ml glass vials hermetically sealed and warmed at 28°C for 1 h. After warming, injections were performed at 180°C. For each sample, three repetitions were performed.

Ten different sensors were used: s1 (WMA-CCTO1), s2 (WMA-US5), s3 (WMA-CCTO2), s4 (WMA-US6), s5 (WMA-CCTO3), s6 (WMA-US1), s7 (WMA-CW1), s8 (WMA-US2), s9 (WMA-CW3), and s10 (WMA-U3). Each sensor is sensible to different kind of volatile molecules i.e., s1 for aromatic, s2 for generic compounds, s3 for aromatic, s4 for hydrogenated, s5 for aromatic-aliphatic, s6 for hydrocarbons, s7 for sulfur, s8 for alcohols, s9 for sulfur chlorides, s10 for hydrocarbons-aliphatic. During the analysis the response of the sensors were monitored at 1 s intervals for an overall time of 95 s at a flow rate of 400 mL/min. The sensor data were expressed as the ratio between signal sensor and minimum signal sensor recorded (data not showed). The signal evaluation was done following the method reported by Sado Kamden et al. (2007), in order to find out which are the most indicative signals for the evaluation of the differences among the samples.

### Statistical analysis

Microvinification were performed in triple. The data obtained are the mean of three independent repetitions. The electronic nose analyses, for each independent experiment, were repeated five times.

The oenological were analyzed by 1-way Anova using the statistical package Statistica for Window (Statsoft Inc. Tulsa, OK). The ability of each parameter to discriminate among the samples was investigated according to the *post-hoc* comparison of the Anova.

For volatile compounds and amino acids the variability coefficient was reported.

The raw data obtained for electronic nose were subjected to principal component analysis (PCA) by using Statistica (Package for Window).

## Results

### Fermentation kinetics and wine analytical profile

In order to evaluate the effects of yeast strain on the physicochemical wine characteristics, Trebbiano musts were inoculated with the 10 strains at level of about 6 Log cfu ml^−1^.

**Figure 1:**
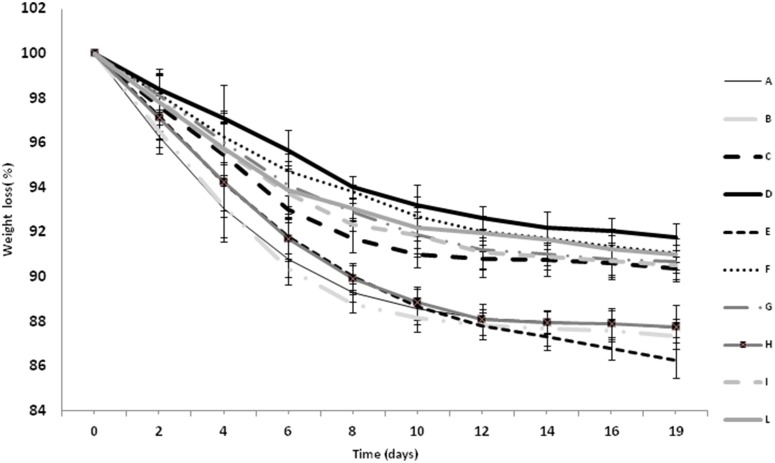
**Sample weight loss during fermentation in relation to the strain used**.

The fermentation kinetics were evaluated measuring the weight loss of musts during fermentation at 18°C, as shown by Figure 1. Data obtained indicated that strains L674 (C), L951(D), M692(F), U5298 (G), 7541(I), and 6644 (L) had similar kinetics, characterized by a reduced amount of fermented sugars, in particular for strain D. On the other hand, strains L234 (A), L288 (B), M630 (E), and H (6944) fermented faster and with a deep sugars consumption. This behavior is confirmed by data of Table 2, where, for yeast A, B, E, and H, the highest alcohol contents are shown. As expected, the yeast strains have produced, in strain dependent way, succinic acid, which ranged from 0.19 (sample fermented with strain D) to 1.74 g l^−1^ (sample fermented with strain A). Total acidity, ranged between 5.10 and 12.0 g l^−1^. Several differences, in strain dependent way, were reported also for the malic and lactic acid (Table 2). A significant decrease of malic acid was observed, comparing to the must, for all the inoculated samples. The lowest decrease in mailc acid concentration were observed in wines produced with the strains H (6944) and A (L234). The decrease of malic acid was not always accompanied by the increase of lactic acid. The pH values ranged between 3.05 and 3.27 according to the strain used.

### Analysis of volatile compounds

The gas-chromatographic analyses permitted the identification of molecules belonging to different chemical classes such as aldehydes, lactones, higher alcohols, esters, short chain fatty acids, and terpenes (Table 3).

**Table 3:** **Volatile molecules (expressed as mg l^−1^) identified by GC-MS/SPME in Trebbiano wines in relation to the strain used**.

	**A**	**B**	**C**	**D**	**E**	**F**	**G**	**H**	**I**	**L**
**ALDEHYDES**										
Acetaldehyde	0.7	1.6	0.4	2.1	1.4	3.0	2.3	1.0	2.3	1.9
Nonanal	0.2	–^*^	–	–	–	–	–	0.1	–	–
										
**KETONS**										
2,3-butanedione	–	0.1	0.1	–	–	–	–	–	–	–
Methylisobuthyl ketone	–	0.2	0.2	0.4	0.1	0.4	0.3	0.1	0.2	0.2
Acetoin	0.1	–	0.4	0.1	0.4	–	0.4	0.2	–	–
Butyrolactone	0.1	–	0.5	0.1	0.8	–	0.1	1.8	0.1	0.1
										
**ALCOHOLS**										
1-propanol	–	0.3	0.3	0.3	0.1	0.3	0.3	0.7	0.4	1.3
Isobutanol	2.8	4.3	4.2	3.9	4.5	5.2	7.3	3.5	6.0	2.8
1-butanol	0.1	0.1	0.1	0.1	0.1	0.1	0.1	0.2	0.1	0.1
Isoamylic alcohol	83.2	80.3	100.5	68.4	85.7	78.1	111.0	93.7	108.3	84.0
3-methyl pentanol	–	0.1	–	–	0.3	0.1	0.2	0.3	0.2	0.3
1-hexanol	7.6	6.4	8.5	4.8	8.0	5.9	5.6	6.7	7.8	7.2
(Z)-3-hexenol	0.2	0.2	0.1	0.0	0.2	0.2	0.2	0.2	0.2	0.2
(E)-3-hexenol	0.3	0.3	0.4	0.2	0.4	0.3	0.3	0.3	0.3	0.3
1-heptanol	0.3	0.4	–	48.8	–	14.9	12.9	–	0.2	–
1-octanol	0.2	0.1	–	0.2	0.2	0.1	0.5	–	0.7	0.2
Nonanol	0.3	0.2	0.1	0.2	0.3	0.1	0.2	0.3	0.2	0.2
Phenylethanol	72.3	43.2	54.6	72.3	52.5	38.6	61.1	81.6	71.6	72.1
Ethylphenol	0.3	0.1	0.5	0.3	0.1	0.2	0.1	–	–	0.7
**ESTERS**										
Ethyl acetate	12.8	7.8	6.3	103.0	12.5	73.4	29.9	11.6	14.7	10.4
Isoamyl acetate	0.9	1.5	0.8	2.1	1.3	1.8	1.5	1.2	1.8	0.9
Ethyil hexanoate	1.7	2.5	2.8	1.0	2.4	1.7	2.4	2.7	3.1	2.2
Hexyl acetate	0.1	0.3	0.2	–	0.1	–	–	0.2	–	0.1
Ethyl octanoate	7.5	4.1	4.6	2.5	6.7	2.4	6.2	5.5	9.0	4.0
Ethyl hydroxy caproate	–	0.2	–	–	–	–	–	–	–	–
Ethyl decanoate	2.4	1.3	1.1	1.3	1.8	0.6	2.1	2.6	3.5	2.1
Diethyl succinate	1.1	1.0	1.9	2.7	1.1	2.4	1.8	2.4	1.9	2.8
Ethyl 9 decenoate	5.3	1.5	1.5	1.3	3.2	0.7	2.4	3.0	3.4	1.9
Ethyl phenyl acetate	0.2	0.2	2.4	0.7	0.1	0.9	0.6	0.2	0.1	0.1
Phenyl acetate	3.1	2.8	2.8	6.4	2.7	2.3	2.2	6.5	4.0	4.9
Ethyl 9 octadecenoate	0.8	–	0.2	0.1	0.3	–	–	0.3	–	–
**TERPENIC ALCOHOLS**										
Linalool	3.2	0.2	2.8	1.7	1.7	0.3	0.3	1.7	0.2	0.6
α-terpineol	1.1	0.1	0.9	0.7	0.6	0.1	0.1	1.2	0.1	0.1
Citronellol	2.7	0.3	2.3	1.1	1.4	0.1	0.2	2.6	0.3	0.3
**ACIDS**										
Acetic acid	13.9	2.7	14.3	20.2	17.7	11.7	12.2	18.1	8.1	8.3
Isobutyric acid	0.2	0.3	0.1	0.7	0.4	0.4	0.8	0.4	0.3	0.2
Decanoic acid	3.9	2.8	4.3	5.2	3.6	1.6	4.4	6.0	3.2	6.5
3-methyl butyric acid	2.2	1.7	1.5	4.6	2.1	2.7	5.5	2.0	1.9	1.8
2,2-dimethyl octanoic acid	–	0.9	1.4	2.5	0.9	0.4	2.8	3.0	1.2	2.4
Octanoic acid	3.4	10.6	24.8	18.4	27.9	19.2	29.0	33.6	17.2	22.2

* *Under the detection limit; The coefficient of variability, defined as the ratio of the standard deviation to the mean, ranged between 5 and 7%*.

Regarding aldehydes, the strain F produced the highest amounts of acetaldehyde, while the strains A and H produced nonanal, having a great sensorial impact. For what concern ketons, quantitative and qualitative differences were observed among the samples, in relation to the strain used. For example, the wine produced with the strains C, E, and H were characterized by great amount of butyrolactone. The strain C, E, and G have produced in wines high amount of acetoin, absent in wines produced by strains B, I, and L. Only the strains B, C, and E produced low amount of 2,3-butanedione.

Great differences were detected among wines in produced alcohols. The strains C, G, and I produced in wines levels higher than 100 mg l^−1^, associated to production of phenylethyl alcohol higher than 50 mg l^−1^. Low amounts of isoamylic alcohols distinguished the sample fermented by strains A, B, D, F, and L. The wine samples H and I did not presented ethylphenol, a molecule of great impact at low concentration.

Regarding esters, high amounts were detected in all the samples, independently on the strain employed. The most presents were ethylacetate and ethylester of medium chain fatty acids such as hexanoic, octanoic, and decanoic acids. Ethylacetate was highly produced by strain D, F, and G. On the contrary, low productions were detected for strains A, B, C, E, H, I, and L. In general, the highest production of esters (excluding ethylacetate) were detected in wines obtained by strain A, H, and I.

Terpenic alcohols, molecules of great sensorial impact, were detected in wines obtained by fermentation of strain A, C, E, and H. These wines showed an accuulation of linalool, α-terpineol, and citronellol.

The tested strains resulted different also in the organic acid release. In particular, acetic, isobutyric, decanoic, 3-methylbutyric, octanoic, and 2,2, dimethyloctanoic acid productions were different in relation to the strain employed. The strongest producers of acetic acid were the strains C, D, E, and H.

### Sulfur compounds

**Figure 2:**
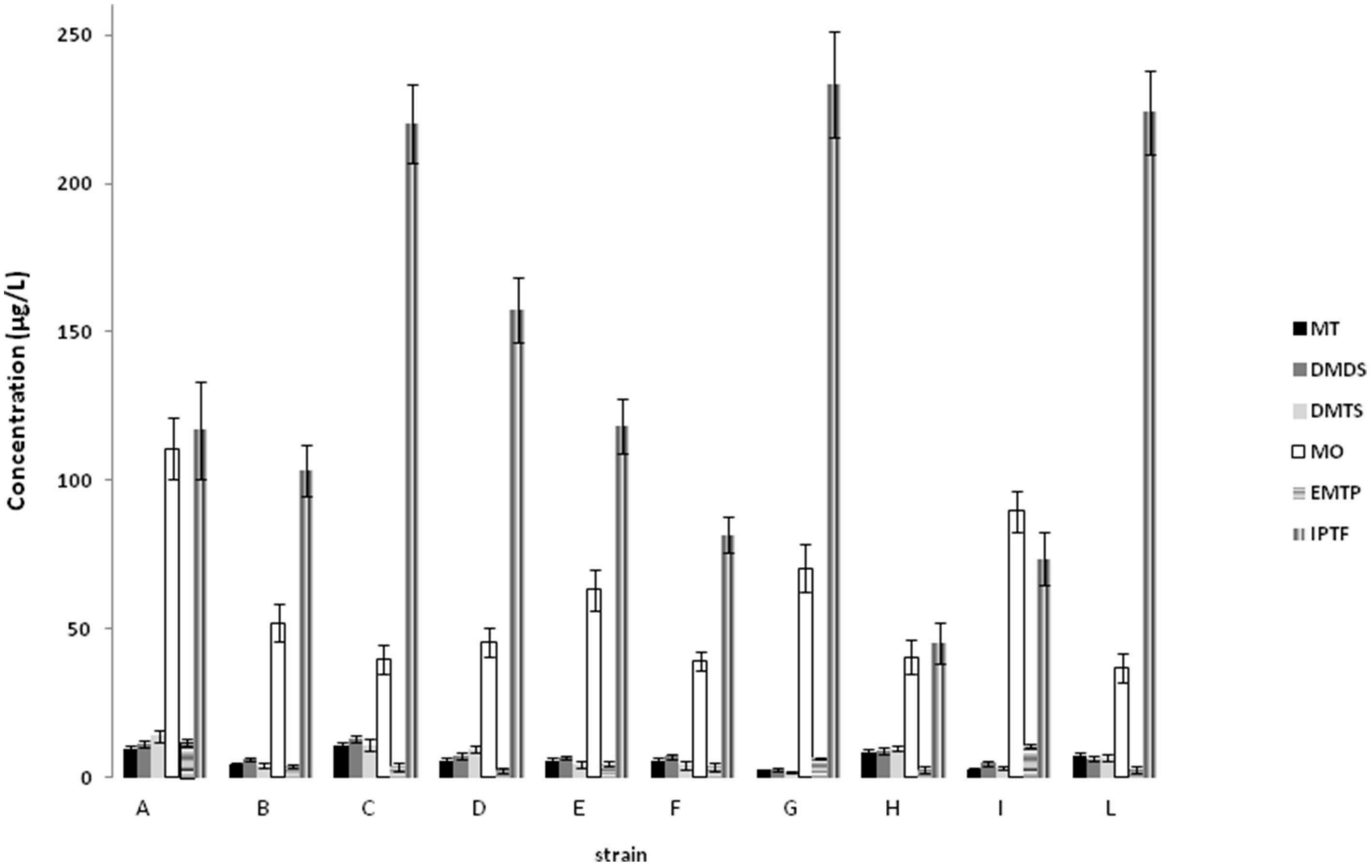
**Sulfur compounds (expressed as μg l^−1^) detected in wine samples in relation to the strain used**. Methantiol (MT), dimethylsulfur (DMS), dimethyldisulfur (DMDS), dimethyltrisulfur (DMTS), 3-methyl-tio-propanol (MO), ethyl 3-methylpropanoato (EMTP), and 4-isopropyltiophenol (IPTF).

The use of a photometric flame detector permitted to detect and quantify in wine samples methantiol (MT), dimethylsulfur (DMS), dimethyldisulfur (DMDS), dimethyltrisulfur (DMTS), 3-methyl-tio-propanol (MO), ethyl 3-methylpropanoate (EMTP), and 4-isopropyltiophenol (IPTF). The detected sulfur compounds, deriving from yeast metabolism, were found in all the samples. All the strains were able to produce high amounts of IPTF (from 45 to 233 μg l^−1^; Figure 2). However, the strains C, G and L produced more than 200 ppb. The strains A, E, I, and G produced high levels of methionol while EMPT was produced at level of 11.82 μg l^−1^ and 10.61 μg l^−1^ in wines produced by strain A and I, respectively. The highest amounts of MT, DMDS, and DMTS were detected in wines deriving from fermentation of the yeasts A and C.

### Amino acid release

Data obtained showed a low level of amino acid content in produced wines, while the must was characterized by high level of all the investigated amino acids (Table 4). Alanine, cystein, methionine (present at low level also in must), proline, leucine, isoleucine, valine, threonine were completely metabolized independent on the considered strain. On the contrary, in wine produced by strain A, with respect to the must, there was an increase of arginine and tryptophan. Arginine was found at low level in wine produced by strain D, E, G, and L.

**Table 4:** **Amino acid content (mg l^−1^) detected in Trebbiano wines in relation to the strain used**.

	**Must**	**A**	**B**	**C**	**D**	**E**	**F**	**G**	**H**	**I**	**L**
Alanine	234.34	1.31	3.48	5.36	–	0.41	0.05	1.21	0.15	1.03	–
Proline	622.81	0.23	0.22	0.4	0.09	0.03	0.05	0.04	0.04	0.12	–
Methionine	1.07	–^*^	–	–	–	–	–	–	–	–	–
Cysteine	1.51	–	–	–	–	–	–	–	–	–	–
Leucine	29.55	1.92	2.53	4.97	–	–	–	–	–	–	–
iso-Leucine	23.28	0.7	0.86	1.64	–	–	–	–	–	–	–
Valine	49.41	3.58	1.42	2.37	–	0.17	0.04	0.45	–	0.95	0.14
Threonine	70.71	–	–	–	–	–	–	–	–	–	–
Ornithine	–	–	–	0.06	–	–	–	–	0.06	–	–
Triptophan	319.3	111.64	20.25	18.1	19.22	42.63	27.37	21.83	22.61	24.33	15.3
Phenyl alanine	5.08	–	1.01	–	–	–	–	0.4	–	–	–
Tyrosine	3.76	0.66	3.25	3.75	–	–	–	0.03	–	–	–
Arginine	207.37	640.04	114.04	216	102.53	165.36	129.41	40.29	192.6	353.13	107.03
Glutamic acid	95.52	5.34	0.59	–	–	–	–	–	–	–	–
γ-Aminobutyric acid	128.43	–	–	–	–	–	–	–	–	–	–

* *Under the detection limit; The coefficient of variability, defined as the ratio of the standard deviation to the mean, ranged between 5 and 7%*.

### Electronic nose

**Figure 3:**
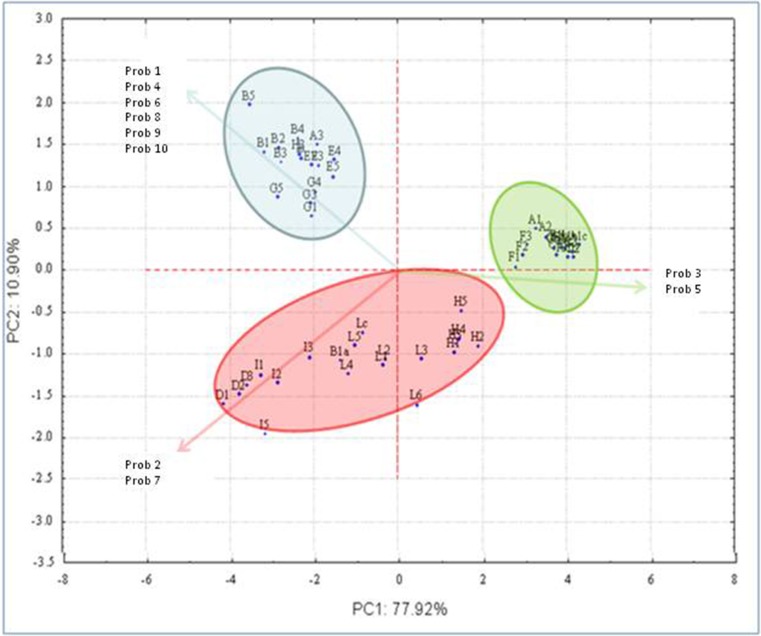
**Principal component analysis loading plot of electronic nose data in relation to the strain used in fermentation**. A (strain L234), B (strain L288), C (strain L674), D (strain L951), E (strain M630), F (strain M692), G (strain U5298), H (strain 6944), I (strain 7541), L (strain 6644).

Because the sensorial profile of a wine is the resulting of volatile and not volatile molecule interaction, the wines were subjected to the electronic nose analysis. The data from electronic nose were obtained by using 10 different probes able to detect different classes of compounds, as reported in Materials and Methods, and give a wine sensory evaluation. The raw data obtained were analyzed by PCA able to discriminate the samples in three different macro-groups in relation to the affinity with the probes used (Figure 3). The first group included wine obtained by strains B, E, G, and H; the second group contained wines from strain D, I, and L, while the wines from strain A, F, and C were grouped together. In particular, the group 2 distinguished for the probes 2 and 7, detecting NO and sulfur compound, respectively. The cluster 3 was formed on the basis of probes 3 and 5, detecting NH_3_ and aromatic compounds and low-polarity aromatic compounds, respectively.

## Discussion

The results of the present work showed that all the *S. cerevisiae* strains were able to drive the fermentation although with different kinetics. As expected, the strains have produced wines characterized by different amounts of succinic acid. On the other hand, the production of this acid, normally absent in the must, is related to yeast metabolism during alcoholic fermentation (Rainieri et al., 2003). Also the difference in total acidity, ranging from 5.1 to 12.65 g l^−1^, can be associated to the strain ability to produce different amount of several acids (succinic, acetic, lactic, and malic acids) but also to the release of different amount of mannoproteins, during the fermentation. The significant decrease of malic acid detected in wine samples, compared to the initial must, can be attributed in the major part of sample to the ability of *S. cerevisiae* strains to degrade malic acid more than malo-lactic fermentation, (Styger et al., 2011). In fact it is well know that *S. cerevisiae* strains are able to degrade or produce malic acid in a strain dependent way. A wide literature have shown that these polymers, produced in strain-dependent way during the yeast growth, fermentation and autolysis, can affect also the tartaric acid concentration and its stability (Caridi, 2006; Palomero et al., 2007).

Until a few decades ago, wine yeasts were selected basically on their ability to quickly transform grape sugars into ethanol, on their resistance to sulfur dioxide and on the low acetic acid production. Actually, their role has been significantly expanded by the advent of modern oenological microbiology and their selection has therefore involved the development of techniques for detecting strains that might improve wines in terms of color, aroma, structure, technological, and also healthy properties. In the present work, in addition to test the yeast fermentation power, also the strain ability to produce wine with characterizing flavor was investigated. In particular, the volatile sulfur compounds production, and in general the volatile molecule profiles produced by starter cultures have a main role in the strain selection and in the product characterization. Some researchers have suggested that these profiles can be regarded as footprints or “aromagrams” and can in the future be used for identification and quality control purposes (Styger et al., 2011). These aromagrams are not only composed of various chemical classes of compounds (alcohols, esters, aldehydes, ketones, acids, and sulfur- and nitrogen-containing compounds), but these compounds have a very wide concentration range in the wine varying between the gram to the nanogram per liter (Bonino et al., 2003). Moreover, it is their ratio which plays an important role in the final wine flavor and taste.

The GC-MS volatile molecules profiles obtained in this work resulted strain dependent and the results are in accordance with Vernocchi et al. (2015) who demonstrated that Trebbiano wines fermented with wild *S. cerevisiae* strains were characterized by proper unique aromatic profiles. Also Mauriello et al. (2009) found that a great variability in volatile molecules produced among the tested wild wine yeasts, emphasizing the potential role of this parameter as trait for starter culture selection. Moreover, Romano et al. (2015) found that volatiles detected by mass spectra techniques represent a strain fingerprinting. Also Tufariello et al. (2014) found that yeast species and, within each species, different strains exhibit wide differences in volatile compound profiles in the production of Negroamaro wines. In this research, for example, the strain F produced the highest amounts of acetaldehyde, while the strains A and H produced nonanal, having a great sensorial impact. The wine produced with the strains C, E, and H were characterized by great amount of butyrolactone. In the obtained wines, also terpenic compounds and esters were found. In general, esters are formed by yeasts during the alcoholic fermentation and they are responsible for the fruity odor, while terpenic and nor-isoprenoid compounds are the most important constituent of the varietal aroma of grapes and confer a flowery odor to the wine (Vararu et al., 2016). In wines obtained by the strains A, C, E, and H linalool, α-terpineol, and citronellol, able to impart citrus and peach flavor notes, were found. In general, these are released in wine also by the yeast ß-glucosidase activities (Pedersen et al., 2003; Fia et al., 2005). By now, numerous works have shown that yeasts involved in vinification processes possess β-glucosidase activity, and this is greater in non-*Saccharomyces* yeast strains than in *S. cerevisiae* ones (Fia et al., 2005). Also volatile esters constitute one of the most important classes of aroma compounds and are largely responsible for the fruity aromas associated with wine and other fermented beverages (Vararu et al., 2016). Their formation differs widely between yeast strains and other external factors such as fermentation temperature, nutrient availability, pH, unsaturated fatty acid/sterol levels, and oxygen levels all playing an important part in determining the end levels of esters in a wine (Lilly et al., 2000). Our data suggested that the highest production of esters (excluding ethylacetate) was detected in wines obtained by strains L284 (A), 6944 (H), and 7541 (I). For example this last strain produced high amount of ethyl hexanoate (whose odor descriptor corresponds to fruit, pineapple), ethyl octanoate (apricot). Also higher alcohols play a fundamental role since they have usually a strong pungent smell. Differently, 2-phenylethanol is an aroma carrier and its presence may contribute to the floral nuance of wines, especially for white wines. The aroma characterized by this compound changes with its oxidation from a rose to a hyacinth bouquet (Duarte et al., 2010). The strains I (7541), A (L284), D (L951), H (6944), L (6644) were able to produces in Trebbiano wines the highest amounts, contributing to positively affect the final aroma. Also sulfur-containing compounds play an important role in wine aroma. Sulfur compounds contribute mainly to unpleasant aromas in wines, although some of them have been reported to have a positive contribution to wine (4-mercapto-4-methyl-2-pentanone, 3-mercaptohexyl acetate, 3-mercapto-l-hexanol,4-mercapto-4-methyl-2-pentanol, and 3-mercapto-3-methyl-l-butanol). In this research, methionol was the heavy sulfur compound present in wines in the highest concentrations with IPTF. Similar results were obtained by Moreira et al. (2010) for monovarietal white wines. According to Falqué et al. (2002), methionol concentration was one of the variables responsible for the differentiation of wines from Loureiro, Dona Branca, and Trajadura cultivars from the Galicia region (Spain). Methionol is produced by yeast from methionine, via deamination, followed by decarboxylation (Ehrlich reaction); the aldehyde thus formed, 3-(methylthio)-1-propanal (methional), is then reduced to the alcohol (methionol) or oxidized to the acid (3-(methylthio)propionic acid). The reaction of methionol with acetic acid yields 3-(methylthio)propyl acetate (Rauhut, 1993). The content of methionol increased considerably in wines with reduction defects (Mestres et al., 2002), contributing odors of potato, cauliflower, and cooked vegetables/cabbage. In our research, the highest amounts of methionol were produced by strain A (110 μg l^−1^), I (89 μg l^−1^), and G (70 μg l^−1^). However, the data of the present research showed that the impact of the sulfur compounds detected is not so strong because in relation and *in equilibrium* with other volatile and not volatile compounds. In fact, the PCA analysis, performed on the data from electronic nose, divided the wine samples only in three homogeneous clusters. On the other hand, this kind of analysis can account the sensory profiles of a wine and reflect the interaction between volatile and not volatile molecules.

## Conclusions

The present work showed that the omic technique adopted (GC/FPD and GC/MS-SPME) can be used as fingerprinting tools and, since they are successfully combinable with those produced by conventional analysis techniques, they can allow to discriminate among the tested strains, in order to select the best candidate in relation to the desiderated wine sensory features. In fact volatile compounds and HVSC, are fundamental for the characterization and definition of the wine sensory properties. The data obtained in this research outline the importance of strain aromagramma in the yeast strain selection for winemaking. In fact, the data contributed to the non-conventional characterization of the employed *S. cerevisiae* strains. In fact, although all the strains showed potential to ferment Trebbiano must, different profiles for volatile and sulfur compounds were identified and fundamental for strain discrimination. Although these preliminary data can useful for the selection of strains in Trebbiano winemaking, further studies regarding other technological features, such as the mannoprotein release and the production of molecule of health importance, such as ethylcarbammate, can be performed. Moreover, additional investigations regarding the genes involved in the sulfur production from the selected yeasts need to be investigated.

## Author contributions

All authors listed, have made substantial, direct and intellectual contribution to the work, and approved it for publication.

### Conflict of interest statement

The authors declare that the research was conducted in the absence of any commercial or financial relationships that could be construed as a potential conflict of interest.
